# Medical students’ experiences with sexual discrimination and perceptions of equal opportunity: a pilot study in Germany

**DOI:** 10.1186/s12909-020-1952-9

**Published:** 2020-02-22

**Authors:** Konstantin Jendretzky, Lukas Boll, Sandra Steffens, Volker Paulmann

**Affiliations:** 1 Dean’s Office – Curriculum Development, Hannover Medical School, Hannover, Germany; 2 Institute for Sociology – Work Group Methods of Empirical Research, Leibniz University, Hannover, Germany; 3 Dean’s Office – Evaluation Office, Hannover Medical School, Carl-Neuberg-Str. 1, 30625 Hannover, Germany

**Keywords:** Medical education, Medicine, Sex, Gender, Equal opportunity, Sexism, Discrimination

## Abstract

**Background:**

Data is available on sexual discrimination and subjective perceptions of equal opportunity in medical education for many countries. Surveys focussing on sexual harassment have not yet been conducted at German medical schools.

**Methods:**

A student initiative surveyed all medical students at the Hannover Medical School (MHH) using an anonymous online questionnaire on equal opportunity and sexual discrimination to identify potential problems in education.

**Results:**

A total of 343 students (15%) participated in the survey. Over 50% reported having either witnessed sexual harassment or experienced it themselves. Female students indicated having experienced sexual harassment three times more often than their male peers; verbal forms of sexual discrimination predominate. These observations and experiences of sexual harassment demonstrated significant influences on many perceptions regarding equal opportunity and equal treatment in the MHH undergraduate medical education at MHH.

**Conclusion:**

This blind spot in medical education in the German-speaking countries should be scrutinized more closely. The experience of sexism in the context of undergraduate medical education, which has negative effects on students, should no longer be ignored in empirical education research.

## Background

Over the past 20 years the percentage of female medical students graduating with medical degrees in Germany has successively increased. It is now just above 60% [1]. Yet, the formation of a critical mass has not accelerated professional advancement in the same way. Women doctors are less often found in leadership positions; they less frequently attain the highest academic posts [2], and differences between men and women exist particularly in terms of their preferences for graduate and post-graduate fields of study [3]. This reinforces the impression of a persisting patriarchal structure in medicine, deeply rooted in pronounced power differentials, student-teacher dependencies and close, often stressful workplace arrangements [4–6]. In fact, male dominance is based on a variety of elements shaping the perspectives and trajectories of the men and women working in this field – sometimes quite visibly, sometimes invisibly as the proverbial “glass ceiling”. One issue that has widely attracted attention in recent years is sexual harassment as a particularly severe type of gender discrimination. Research on various forms of sexual harassment has demonstrated that this is a widespread phenomenon in academia and particularly in medicine [7]. Sexual harassment itself ranges from subtle forms to criminal acts. Schneider and Philips [8] divide it into four categories: a) gender harassment which encompasses insulting or degrading sexist remarks; b) unwanted sexual attention, including touching or requests for dates after a person has refused; c) sexual bribery/coercion; d) attempted sexual assault [8]. These categories align with incidents of gender discrimination that were described by students themselves [9]. What the different manifestations have in common is that they serve to maintain a given power structure and “to remind the powerless of their position in the hierarchy” [8].

Qualitative research has retraced the “gender learning curve” women encounter in medical education [10, 11] and the internalisation of gender roles [12] that is often produced by the repeated confrontation with patterns of male dominance. Gender research has extensively reflected on the boundaries and immanent roles men and women are confronted with and has provided different analytical tools [13–16]. Overcoming the classical categorical differentiation between two existing sexes has opened up space for perspectives on gender that reveal the many pathways and performances which are influenced more by culture than by biology. In recent years, stress has been placed on the dynamic and often fluid aspects of gender ascriptions. This interpretation of gender in an interactive approach has led Hagemann-White to the insight that “Gender *is* a relation “[16], meaning that identities are shaped in everyday rituals and confrontations. In contrast to other disciplines, these refined definitions and gender-related issues have long been sidelined in academic medicine and in medical education research [17, 18]. Verdonk even revealed the strong resistance gender-sensitive research sometimes faces from medical faculties [19]**.**

After a controversial media debate on sexual discrimination in the aftermath of the # MeToo movement, which has also reached the medical field [6], a student initiative formed at the Hannover Medical School (MHH) to discuss the influence of patriarchal structures at the medical school. Unlike other medical schools in Germany, MHH only offers study programmes in medicine, dentistry, public health and biomedicine. Founded in 1965 on the outskirts of Hannover and with the university hospital located on the campus, MHH had already implemented progressive curricular approaches with student support and now currently offers a model curriculum [20]. The percentage of female medical students has increased to 70% in the past 10–15 years. In Germany, as in many other countries, there is equal rights legislation [AGG] in place that has also been incorporated into many medical degree programs. In 2017, MHH published guidelines on how to deal with sexual discrimination [21]. Unfortunately, the mere existence of written policies or a legal framework does not mean that discrimination stops when the power imbalances persist in the everyday routines of men and women [12].

As the initiative went on to organize a panel discussion on sexualised discrimination and violence at MHH in 2018, it became clear that there is a peculiar lack of data for Germany with regard to published evaluations on sexual discrimination in medical education. Even periodic cross-discipline surveys and questionnaires, such as the Student Survey (*Studierendensurvey)* administered by the University of Constance or the data collected by the *Deutsches Zentrum für Hochschul- und Wissenschaftsforschung* (DZHW), are designed to address the quality of academic programs and student satisfaction with learning environments at a national level and do not explicitly cover the topic of sexual discrimination. There is only one study that analyses the prevalence of various forms of negative experiences in undergraduate medical education, with sexual harassment among them [22]. There, only 8% of the students reported having experienced sexual harassment. This blind spot suggests that sexism in German medical education does not exist. However, reports from different countries illustrate that sexual harassment in medicine is a global and multifaceted problem: in addition to other forms of ostracism, there is a high prevalence of sexual discrimination in medical education that affects women at a higher rate than men [4, 23–29]. In their meta-analysis, Fnais et al. [23] uncovered that, on average, 54% of those surveyed reported experiences of sexual discrimination within the scope of their medical education. The impact on those who are affected can be severe and persistent: a reduced well-being, lower self-esteem, a lack of motivation and even depression among physicians are reported [4, 5, 24, 25, 27, 28]. With regard to students and junior professionals, other studies have demonstrated that mistreatment, belittlement, disrespectful behaviour and sexual harassment have a negative impact on overall satisfaction with the educational experience [5, 30, 31]**.** Deidealization and a decreased motivation to become a doctor may follow [4, 32]**.** In addition to the individual consequences, harassment also affects patients since unwell physicians do not deliver high-quality healthcare [33] and are more likely to quit their hospital positions [24] or change specialty [34]. Mistreatment is regularly analysed with regard to its prevalence in different subgroups or to shed light on related issues, e. g. mechanisms of silencing the problem or individual or institutional coping strategies; the majority of incidents indeed remain unreported [25, 35]. Little is known about how students who have witnessed or experienced sexual harassment in their learning environment perceive career opportunities for men and women in medicine.

In light of this and as part of a pilot study, the initiative surveyed medical students at MHH regarding their experiences involving sexual discrimination and their views on equal opportunity. Students’ perspectives on the existing support structures at MHH in cases of sexual discrimination were also surveyed. This explorative approach follows two analytical lines of inquiry. First, witnessing and experiencing sexual discrimination were evaluated with a focus on comparing female and male perspectives; and second, the extent to which these experiences are connected with student satisfaction, perceived professional opportunities and professional goals. Against the background of the reviewed literature, we wanted to answer the following research questions:

- Are female students still the main target of gender discrimination in medical education, regardless of the rising percentage of women over the last decades?
- Are students, women and men alike, who were confronted with sexual discrimination less satisfied with their course of studies and did they rate their professional chances more sceptically with regard to gender equality in medicine?

## Methods

### Development of the questionnaire

Data collection was conceived as part of the preparation of a podium discussion held in May 2018 entitled “Sexism ≠ MHH”. MHH Student Council (*AStA*) representatives and other politically active university groups initiated an online survey of all undergraduate medical students. To ensure the quality of the questionnaire, the Equal Opportunity Officer, a lecturer in Medical Sociology, and an employee in the Evaluation Office (VP) monitored the process of reviewing the questions. Questionnaires on this topic previously used and published in Anglo-Saxon countries served as a basis for creating a customized questionnaire [6] and were adapted to fit the context. Relevant aspects of sexual discrimination were discussed and documented in four meetings using reports of personal experience; the questions were then formulated concretely. The resulting topics were as follows:

- Overall student satisfaction
- General responses to questions regarding the professional development of male and female physicians and perceptions of equal opportunity;
- Personal experiences with sexual harassment that are addressed separately according to the predefined categories of witnessing harassment or experiencing it;
- Perception of opportunities for receiving information and counselling at MHH;
- Socio-demographic details.

A detailed overview of the items and the respective answer categories is provided in Table 2. For each item there was a box for “abstention” that was labelled “not sure” or “I cannot answer”.

Sexual harassment was assumed to take place if at least one of the following seven situations was described: I. sexually degrading comments; II. sexually degrading gestures; III. unwanted sexual contact / certain physical touching; IV. unwelcome and visible display of pornographic material; V. unwanted and persistent advances; VI. subtle sexual bribery, or VII. sexual assault.

The identification of the different categories of sexual harassment followed examples provided in the MHH guidelines on how to handle sexual discrimination and violence [21], whereby we aimed to use clear and comprehensible definitions to describe the routinely observed behaviours. The category “subtle sexual bribery” [6] which includes the use of stereotypical gender roles in everyday situations was added and brief examples were given in the questionnaire, (e. g. gender-specific terms such as “sweetheart” or gender-specific behaviours such as “needing a strong man”). In addition to the kind of harassment, the group of persons involved was also indentified (students, teachers, unknown person/other). In addition to the straightforward questions, information was also asked for in an open-ended text field regarding the gender of the persons involved and against whom the harassment was directed. A detailed description of the incident was not explicitly asked for, but was nonetheless optional.

### Data collection

Data collection took place in April 2018 by means of an online survey. All medical students who were enrolled at the time were invited to participate via their university email accounts (*n* = 2114). Two reminders were sent out before the survey was concluded at the beginning of May; the assignment of a personal transaction number (TAN) prevented the submission of duplicate questionnaires or the participation of third parties. The time needed to take the survey was approximately 15–20 min. A small number of dental students were included by mistake (*n* = 24). An analysis of the TANs revealed that only four dental students participated in the study. Due to anonymity, their answers could not be excluded.

### Statistical analysis

Descriptive analyses and comparisons of means at the group level are used to present the results for the closed-ended items. For the comparisons, a t-test was performed to compare male and female students in order to check existing differences for significance. The distributions of categorical variables were tested for significant differences using the χ^2^-test. If variables had less than five cases in one or more answer categories, categories were summed up. When this was not reasonable, Fisher’s exact test was used. A value of *p* < 0.05 indicated statistical significance. In order to detect the connection between sexual harassment and other variables, the reported incidents of sexual harassment were transformed into a variable with three values to convey whether: 1.) any kind of harassment was witnessed, 2.) harassment was only witnessed, or was 3.) witnessed and experienced. This variable was used for subgroup analyses in addition to gender stratification and Anova was applied. All analyses were carried out using Stata 13.1. In addition, the open-ended responses that more concretely described the observed incidents and personal experiences were analysed for content. Categories were defined in advance and independently applied to the material by two of the authors (KJ; LB).

## Results

### Participants

Of the students contacted, *n* = 343 completed the questionnaire. The response rate was approximately 15%. The ratio between men and women was only slightly skewed in the statistical sample (percentage of women/men in the MHH student population: 73% vs. 27%; in the sample: 69.7% vs. 29.1%; there was no indication of gender for 1.2% of respondents). The gender category “other” was not marked. The distribution between first to sixth academic year was between 11% (sixth) and 20% (third/fourth year students). Relevant gender-specific differences in the socio-demographic data only exist with regard to the final academic degree sought: in the sample, men identified the higher goals (professorship/post-doctoral qualification) twice as often as women. All of the socio-demographic data of the participants are presented in Table 1.

**Table 1:** Socio-demographic details for the surveyed students

		n	Mean	Percent	Standard deviation
Gender	Female	237	–	69.7	–
	Male	99	–	29.1	–
	No indication	4	–	1.2	–
Country of birth	Total	330		90.3 / 9.7	
0 = in Germany; 1 = not in Germany	Female	210 / 23		89.0 / 9.7	
	Male	88 / 9		89.9 / 9.2	
Age	Total	331	23,3		± 3.70
	Female	232	23.3		± 3.82
	Male	99	23.5		± 3.44
Academic degree sought (Multiple responses possible)	Total	336			
Female					
1 = Medical doctor		187		78.9	
2 = Specialty attained (Facharzt)		173		73.0	
3 = Post-doctorate (Habilitation)		29		12.2	
4 = Professor		18		7.6	
Male					
1 = Medical doctor		85		85.9	
2 = Specialty attained (Facharzt)		67		67.7	
3 = Post-doctorate (Habilitation)		23		23.2	
4 = Professor		17		17.2	

### Gender differences

#### Sexual harassment

**Fig. 1:**
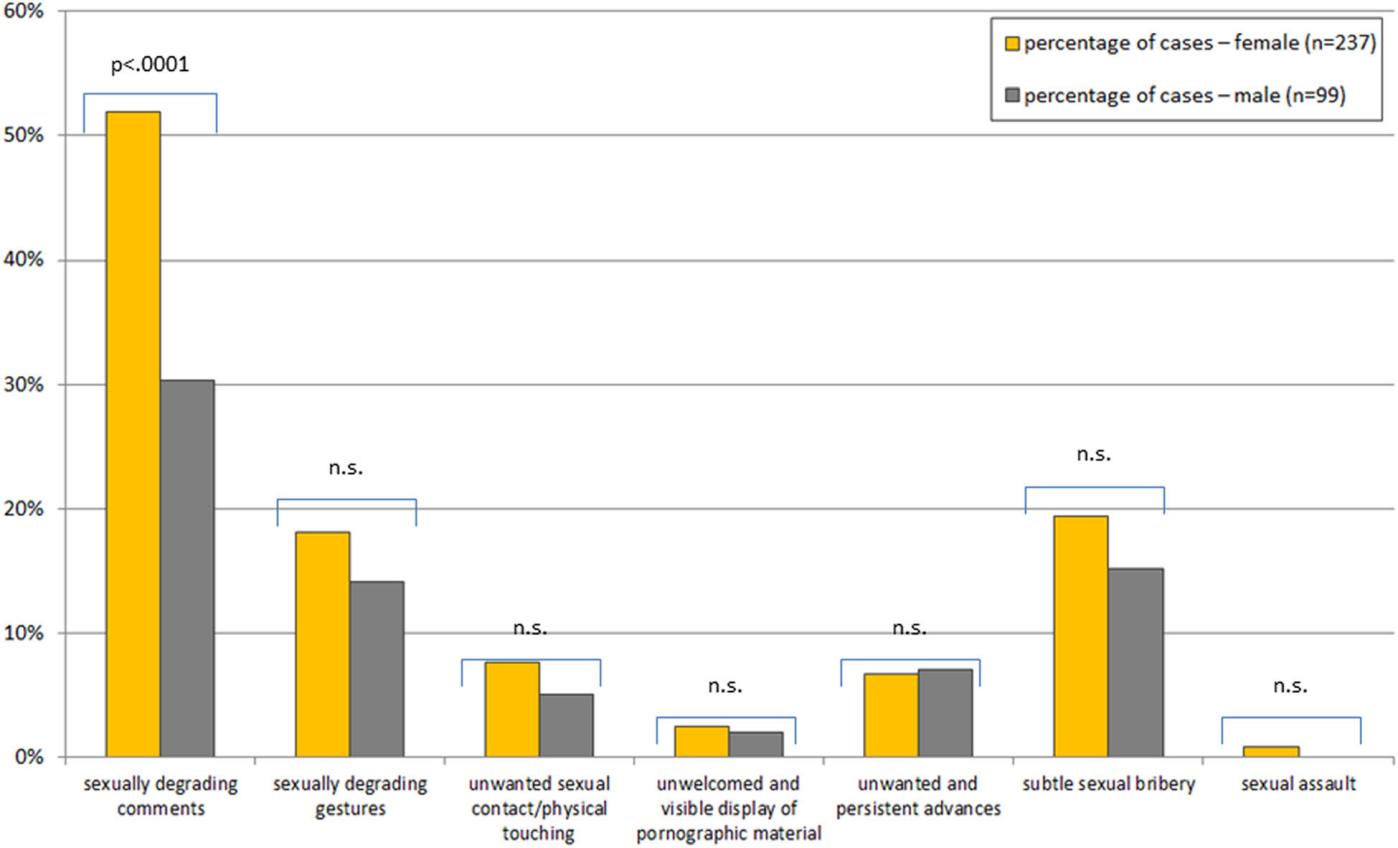
Percentage and forms of witnessed incidents of sexual harassment during undergraduate medical education according to sex (and results of the χ2-test for significance). 53.9% (59.1% of women and 41.4% of men) of students have observed at least one form of sexual harassment

**Fig. 2:**
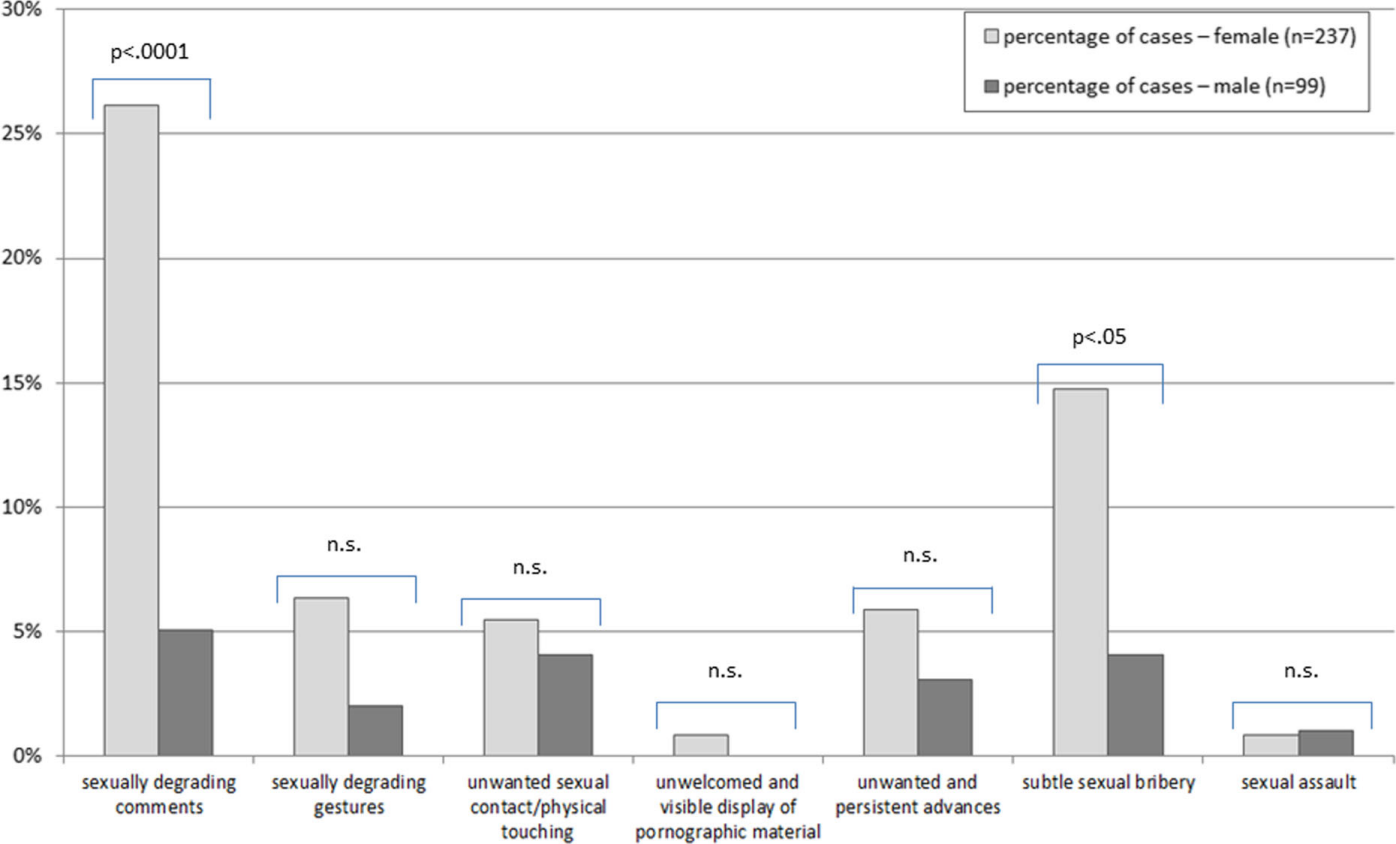
Percentage and forms of directly experienced sexual harassment during undergraduate medical education according to sex (and results of the χ2-test for significance). 29.2% (36.3% of women and 12.1% of men) of all students have experienced at least one form of sexual harassment

The observation and experience of sexual harassment show significant differences between female and male students. A total of 53.1% (*n* = 182 students; 59.1% of women and 41.4% of men; χ2(1) = 8.8, *p* ≤ .05) indicated they had observed at least one form of sexual harassment (Fig. 1). Furthermore, 29.2% (*n* = 99 students; 36.3% of the women and 12.1% of the men; χ2(1) = 19.7, *p* ≤ .001) have experienced at least one form of sexual harassment (Fig. 2). The share of students who indicate that they have neither witnessed nor experienced any form of sexual harassment at MHH drops from 54% in the first academic year to 28% in the final year. Sexually degrading comments are the most frequently reported type of harassment regardless of gender.

#### Professional development for physicians and perceptions of equal opportunity

With regard to the perceived equality, women rate their chances significantly worse than men. 88% of the women believe that there are better opportunities for advancement at MHH for men, but only 58% of the men agree with this statement (*M* 1.8 vs. 2.4; *T* = 7.1, *df* = 293, *p* ≤ .001). Moreover, 92% of the female students in general believe that having a child affects career advancement in medicine more negatively for women, while only 66% of the male students share this belief. Only 3% of the women and 11% of the men agree with the statement that children do not have a negative influence on a career in medicine. In addition, about 70% of the women state that they are concerned about becoming a parent during training (completely agree/agree), whereas only 32% of their male peers are concerned (*M* 3.6 vs 2.4; *T* = 7.1, *df* = 316, *p* ≤ .001).

When considering later career paths, 44 women (18.9%) stated that they had ruled out a medical specialty they originally aimed at due to their gender. In contrast, only two men (2.0%) made this statement. When asked which specialty, almost all women (*n* = 42) reported abandoning the pursuit of a surgical specialty. 48% (*n* = 17) of this subgroup of women reported that they witnessed and experienced harassment, whereas only 29% of the women who had not changed their favoured specialty reported harassment (χ2(1) = 4.912, *p* ≤ .05). *N* = 15 of the n = 42 women who ruled out surgery also reported unequal treatment on surgical wards.

In line with these findings, agreement with the general statement “no one is at a disadvantage at MHH due to gender” differs significantly between women and men (*M* 2,80 vs 3,1; T = − 2.4, df = 316, *p* ≤ .05), as does the “impression that I have been treated more poorly during my studies at MHH due to my gender” (*M* 1,8 vs 1,5; T = 2.1, df = 310, *p* ≤ .05). Yet, the latter statement that refers to the individual dimension is rejected by the majority of men and women alike.

Interestingly, the overall satisfaction with the academic program at MHH does not show significant differences between women and men (*M* 3.5 vs. 3.5; 1 = very dissatisfied <> 5 = very satisfied).

#### Observations of unequal treatment in teaching situations

**Fig. 3:**
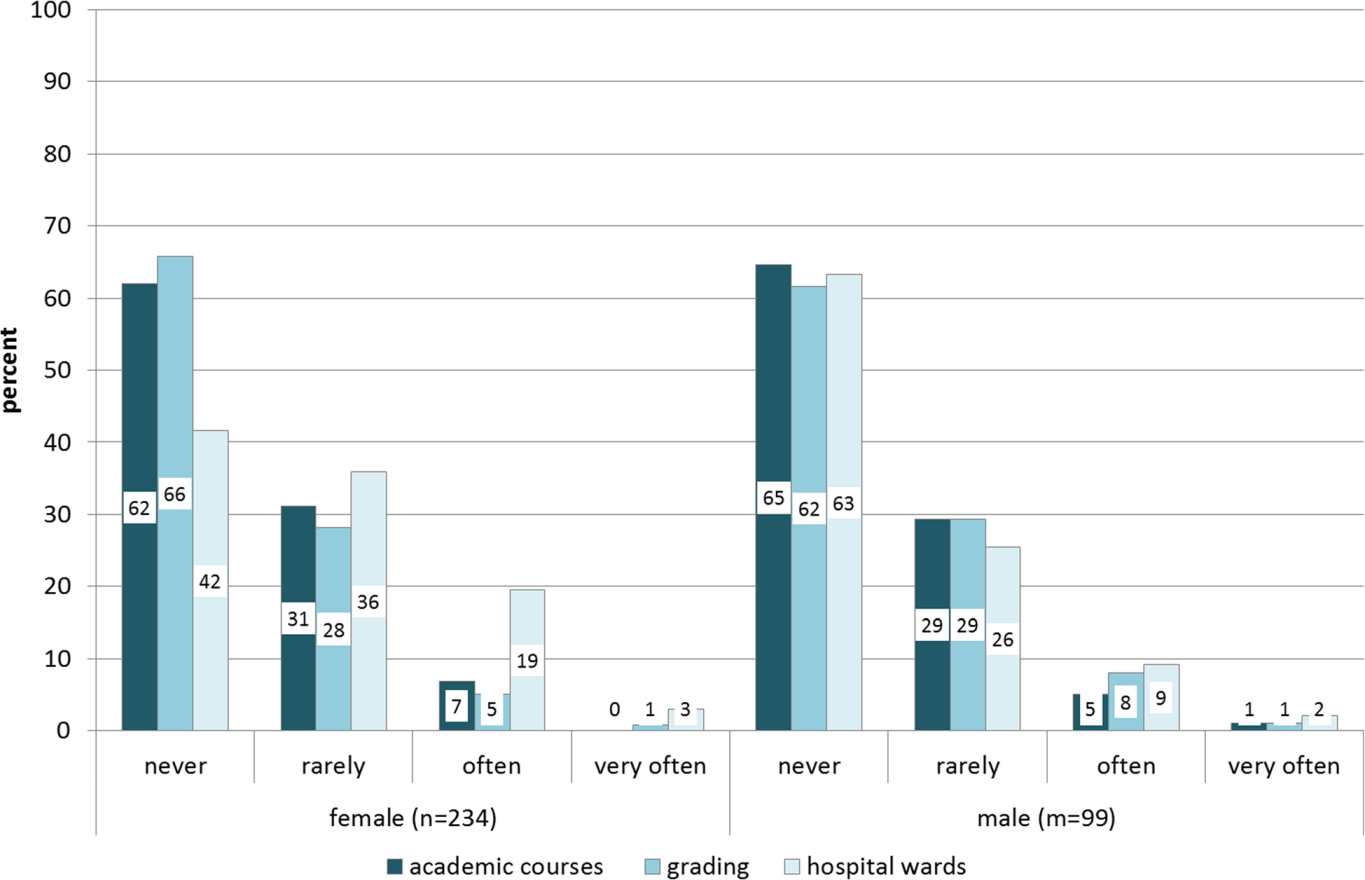
Over the course of my studies I have observed unequal treatment in courses/ discriminatory grading/ unequal treatment on hospital wards

Significantly more female than male students perceived gender-related unequal treatment on hospital wards. About 60% (*n* = 135) of the women reported that they were confronted with such experiences at least “rarely”, “often” or “very often” but only 37% (*n* = 36) of the men indicated similar experiences (χ2(2) = 13.6, *p* ≤ .001). When asked in what medical specialty the unequal treatment on wards took place, about 60% (*n* = 69) of the women and 44% (*n* = 15) of the men indicated that surgery was involved. In contrast, academic courses and grading students did not differ significantly and incidents were generally more rarely reported (Fig. 3).

#### Dealing with and measures against sexual harassment

All questions addressing measures against sexual harassment show a significant gender gap (Table 2). About 65% of the female students think that there should be more discussions about sexual harassment (male: 42,6%), and 50% (male: 26%) would like to see more opportunities for counselling at MHH. Only 25% of the women (men: 40%) answered that they know of the resources at MHH to provide assistance.

**Table 2:** List of single-choice and likert-scale items

n and % for cathegorial variables; mean and standard deviation (sd) for likert-scale variables^a^		n (%) for each category	n (&abstention)^b^	mean	sd	*p*-value^c^
*How satisfied are you as a student at MHH?*	**total**		328 (8)	3.58	± 1.21	
1= very dissatisfied <> 5 = very satisfied	female		233 (4)	3.56	± 1.23	0.6183
	male		95 (4)	3.63	± 1.17	
*No one is at a disadvantage at MHH due to gender.*	**total**		318 (18)	2.9	± 1.21	
1= completely disagree <> 5 = completely agree	female		225 (12)	2.8	± 1.06	0.0187
	male		93 (6)	3.13	± 1.24	
*There are better opportunities for advancement at MHH based on gender. Those having an advantage are…*	**total**		301 (42)	1.97	± 0.85	
1= men <> 5= women	female		210 (27)	1.8	± 0.77	0.0000
	male		85 (14)	2.37	± 0.90	
*Due to my gender I turned a specialty down that I originally favoured.*	**total**	1: 46 (13.9); 2: 212 (63.9); 74 (22.3)				
1= yes; 2=no; 3=not sure	female	1: 44 (18.9); 2: 123 (52.8); 3:66 (28.3)	233 (4)			
	male	1: 2 (2.0); 2: 89 (89.9); 3: 8 (8.1)	99 (0)			
*Having a child during education holds disadvantages for professional careers in medicine especially for:*	**total**	1: 2 (0.6)2: 280 (84.1)3: 18 (5.4)4: 33 (9.9)	333 (3)			
1= men; 2=women; 3=a child does not disadvantage; 4=not sure	female	1: 0 (0); 2: 215 (91.9); 3: 7 (3); 4: 12 (5.1)	234 (3)			0.0000
	male	1: 2 (2); 2: 65 (65.7); 3: 11 (11.1); 4: 21 (21.2)	99 (0)			
*I am concerned that becoming a parent during my medical studies would compromise my future career chances.*	**total**		318 (18)	3.28	± 1.51	
1= completely disagree <> 5 = completely agree	female		228 (9)	3.63	± 1.39	0.0000
	male		90 (9)	2.39	± 1.44	
*Over the course of my studies I have observed unequal treatment in courses due to my gender.*	**total**	1: 209 (62.8); 2: 102 (30.6); 3: 21 (6.3); 4: 1 (0.3)	333 (3)			
1 = never: 2 = rarely; 3 = often; 4 = very often	female	1: 145 (62.0); 2: 73 (31.2); 3: 16 (6.8); 4: 0	234 (3)			0.436^d^
	male	1: 64 (64.6); 2: 29 (29.3); 3: 5 (5.1); 4: 1 (1.0)	99 (0)			
*Over the course of my studies I have observed discriminatory grading due to my gender.*	**total**	1: 215 (64.6); 2: 95 (28.6); 3: 20 (6.0); 4: 3 (0.9)	333 (3)			
1 = never: 2 = rarely; 3 = often; 4 = very often	female	1: 154 (65.8); 2: 66 (28.8); 3: 12 (5.1); 4: 2 (0.9)	234 (3)			0.796^d^
	male	1: 61 (61.6); 2: 29 (29.3); 3: 8 (8.1); 4: 1 (1.0)	99 (0)			
*Over the course of my studies I have observed unequal treatment on wards due to my gender.*	**total**	1: 158 (48.0); 2: 108 (32.8); 3: 54 (16.4); 4: 9 (2.7)	329 (7)			
1 = never: 2 = rarely; 3 = often; 4 = very often	female	1: 96 (41.6); 2: 83 (35.9); 3: 45 (19.5); 4: 7 (3.0)	231 (6)			0.003^d^
	male	1: 62 (63.3); 2: 25 (25.5); 3: 9 (9.2); 4: 2 (2.0)	98 (1)			
*I have the impression that I have been treated more poorly during my studies at MHH due to my gender.*	**total**		295 (41)	1.69	± 0.97	
1= completely disagree <> 5 = completely agree	female		210 (27)	1.77	± 0.96	0.0360
	male		85 (14)	1.51	± 1.00	
*I have the impression that it is easier to apply for doctoral theses for the following gender:*	**total**		196 (140)	2.96	± 0.83	
1 = rather men; 5 = rather women	female		124 (113)	2.8	± 0.81	0.0002
	male		72 (27)	3.25	± 0.80	
*I have the impression that when applying for scholarships…are preferred.*	**total**		190 (146)	3.2	± 0.88	
1 = rather men; 5 = rather women	female		118 (119)	2.97	± 0.83	0.0000
	male		72 (27)	3.57	± 0.84	
*In your opinion, is sexual harassment spoken about too little or too much at MHH?*	**total**		307 (29)	2.3	± 1.05	
1 = too little <> 5 = too much	female		218 (19)	2.15	± 0.96	0.0001
	male		89 (10)	2.67	± 1.16	
*If you were to witness or experience an instance of sexual harassment, are you aware of the units and people at MHH meant to provide assistance?*	**total**	0: 79 (30.5)1: 180 (69.5)	259 (82)			
0 = yes; 1= no	female	0: 45 (24.9); 1: 136 (75.1)	233 (4)			0.0030
	male	0: 34 (43.6); 1: 44 (56.4)	97 (2)			
*In my opinion, the existing MHH programs for victims of sexual harassment are sufficient.*	**total**		150 (186)	2.99	± 1.17	
1= completely disagree <> 5 = completely agree	female		106 (131)	2.86	± 1.11	0.0369
	male		44 (55)	3.3	± 1.27	
*Would you like to see more options to receive information and for counseling at MHH in connection with sexual harassment?*	**total**	0: 140 (62.2)1: 85 (37.8)	225 (111)			
0 = yes; 1= no	female	0: 114 (74.5); 1: 39 (25.5)	153 (84)			0.0000
	male	0: 26 (36.1); 1: 46 (63.9)	72 (27)			

^a^reported incidents of witnessed and experienced sexual harassment are depicted in figure 1 & figure 2

^b^different case numbers result from the fact that either the item was not answered or gender was not indicated

^c^underlined values indicate statistical significance

^d^Fischer's exact test

#### Open-ended responses concerning sexual harassment

Overall, 191 of those surveyed included additional information about the incidents of sexual harassment. It was possible to analyse 120 comments on observed incidents and 71 on personal experiences by statistically counting these non-standardised data. The sex of the harasser and the affected person were recorded (male, female, and/or patient [without specification of sex]). Since reference was sometimes made to separate incidents, it was theoretically possible that data was available for all categories. In addition to gender, the extent to which a professional hierarchy was present was also noted, meaning the mention of higher-ranking male or female physicians, male or female professors, etc. All in all, in around 80% of cases men instigated the harassment for which details were given – consistent among male and female students. However, women students more often identified the affected person as female (in 84% of descriptions); male students identified females as the affected party in approximately 60% of the observed cases and in around 35% of cases men were reported as having been harassed. In nearly all cases the harassment targeted the opposite sex. Around 12% of students also reported having observed harassment coming from patients.

The descriptions of personal experiences of harassment can only be summarized from the perspective of female students (*n* = 17) since an analysis of the descriptions given by male students is unreliable given the low number of cases (*n* = 4). In this small sample nearly all female students report that they have been harassed by a male, and hierarchy plays a role, too: in half of the reports a higher-ranking person is the source of the harassment.

#### Subgroup analyses

Subgroup analyses were conducted to test the relationship between witnessed or experienced gender discrimination and matters of equal opportunity. Separate one-way Anova were applied because the distribution of data shows that within each gender category sexual harassment was reported to a different extent. Three subgroups were formed for women and men, respectively, based on the reported incidents of sexual harassment:

- Group 1: sexual harassment **was not** witnessed **and not** experienced,
- Group 2: sexual harassment **was** witnessed **but not** experienced,
- Group 3: sexual harassment **was** witnessed **and** experienced.

In rare cases (4% women; 3% men) there were reports of harassment experiences but no observations. These students were allocated to group 3. The results show that among men and women differences exist with regard to the assessment of equal opportunity: the more pervasive the harassment was perceived to be, the more sceptically equality-related items were rated (Table 3). There are also different patterns within gender. Female students who witnessed and experienced sexual harassment rated aspects of gender equality significantly more negatively than those who had no personal experience. And those who reported observations are more critical than their female peers who had not. Among male students, the subgroup that reported no observations or experiences is also the most “positive” one, but the other two subgroups differ only slightly from each other. Again, the overall satisfaction as a student at MHH does not show significant differences, neither for female nor for male students.

**Table 3:** Subgroups of female and male students and their perceptions of equal chances in medicine

	Female							Male						
	*neither witnessed nor experienced*		*only witnessed*		*witnessed and experienced*		*p*	*neither witnessed nor experienced*		*only witnessed*		*witnessed and experienced*		*p*
	mean / sd	n	mean / sd	n	mean / sd	n		mean / sd	n	mean / sd	n	mean / sd	n	
*How satisfied are you as a student at MHH? (1= very dissatisfied <> 5 = very satisfied)*	3,7 ± 1.2	87	3,4 ± 1.3	64	3,6 ± 1.2	82	0.325	3,7 ± 1.2	52	3,6 ± 1.1	32	3,3 ± 1.1	11	0.435
*No one is at a disadvantage at MHH due to gender (1= completely disagree <> 5 = completely agree)*	3,2 ± 1	80	2,7 ± 2.5	60	2,5 ± 1	85	0.000	3,5 ± 1.1	54	2,6 ± 1.3	29	2,8 ± 1.4	10	0.007
*I am concerned that becoming a parent during my medical studies would compromise my future career chances (1= completely disagree <> 5 = completely agree)*	3,3 ± 1.5	85	3,6 ± 1.3	61	4 ± 1.2	82	0.002	2,2 ± 1.4	52	2,6 ± 1.4	28	2,7 ± 1.8	10	0.393
*I have the impression that I have been treated more poorly during my studies at MHH due to my gender (1= completely disagree <> 5 = completely agree)*	1,4 ± 0.7	84	1,7 ± 0.8	56	2,2 ± 1.1	82	0.000	1,3 ± 0.8	52	1,7 ± 1.9	28	1,9 ± 1.3	10	0.107

## Discussion

To the best of our knowledge, this is the first survey focussing on sexual discrimination as a form of mistreatment in the context of medical undergraduate education in Germany. Gagyor et al. [22] accentuated a large spectrum of negative experiences with sexual harassment among others. It is surprising that in this context only 8% of the students reported sexual harassment as a negatively connoted incident. Based on the data presented here, it is possible to estimate the extent of sexual discrimination from a different perspective and to gauge the perceptions of equal opportunity in the context of undergraduate medical education at a German university. Over 50% of the participating students described either having personally witnessed or experienced sexual harassment. The risk of personally experiencing sexual harassment was three-fold higher for female students. The prevalence of sexual harassment reported in our study is comparable to the prevalence presented in the large-scale meta-analysis by Fnais et al. [23], in which percentages of over 90% are reported depending upon the timeframe, place of investigation and definitions [36].

These witnessed or directly experienced incidents of sexual harassment showed significant influences on many perceptions regarding equal opportunity and unequal treatment in the undergraduate medical program at MHH. The results also indicate that the “intensity” of the confrontations with harassment has an impact. Students who reported observations *and* experiences are the most sceptical or disenchanted. This effect is partly visible in men, too. This is important because categorical thinking often underestimates diversity within men and women [16]. Although our data indicates a connection between sexual harassment and perceptions of equal opportunities, the essence of this link is not quite clear. Do experiences that students report have an impact on their perceptions of equal opportunities in medicine in general, as an “eye-opening” experience? Or is an existing critical awareness of gender relations associated with a raised sensitivity to inequality in daily routines which fellow students perceive as “normal” and consequently do not report as a form of harassment? This could partly serve as an explanation why about 25% of the students do not report gender discrimination over the course of their medical education at all. This is a topic that deserves to be scrutinized in future research. In a study on career decisions by medical students, Buddeberg-Fischer et al. have demonstrated that women usually anticipate the compatibility of family and career goals even prior to making the actual choice [37]. As a consequence, paths that appear attractive in terms of field and specialty are ruled out. This was also confirmed by our survey. Women have stronger concerns than men about the negative effects of children on their careers. They also rate their chances for advancement and successfully combining family and career significantly lower than men do. Women less often aim for the position of chief physician or for post-doctoral academic qualifications. These results are corroborated by other studies that demonstrate the differing ambitions of men and women in regard to prestigious positions in the healthcare system [3].

It is interesting that virtually all women (*n* = 44) who indicated having ruled out a specialty they had originally aspired to stated that a surgical discipline was involved. Even though our survey did not explicitly ask for the reasons why surgery was abandoned, the women in this subgroup reported sexual harassment at a higher percentage than their fellow students. Other studies have also been able to show that discriminatory behaviour towards women is reported in surgical settings more frequently than average [9]. When viewed against a backdrop that includes a shortage of medical professionals in surgical specialties, the need for change appears even more urgent.

However, limiting the problem of sexual harassment and discrimination to individual medical fields clearly shortchanges this issue. Unequal treatment has occurred in lectures and seminars, in grading practices, and on hospital wards during the final practical year of medical school; it has occurred in both the pre-clinical and clinical phases of undergraduate medical study. The main focus must therefore be placed on the educational environment overall and on the relationship between teachers and students. Efforts to improve the learning environment could lead to profound effects since medical role models have great influence – positive and negative – on future career paths, particularly in the clinical field [9]. Thus, sexual discrimination can have lasting effects, particularly when it is unconsciously incorporated, for today’s students are tomorrow’s teachers [4].

Our data also clearly indicate that the initiators of sexual discrimination are for the most part men and that their comments and behaviour usually target women. Usually, these men occupy a more senior position in the hierarchy of medical education as teachers or superiors. This power structure complicates effective defence and makes it more difficult. Some studies have determined that experiences of discrimination are often not reported at all [12, 24, 38]. In this regard, the availability of people to whom the affected party can turn is of extreme importance. The survey results show that in cases of sexual harassment around half of the students are not aware of the appointed resources and people at MHH who are there to provide assistance. The group making these statements is primarily composed of students who have had been exposed to incidents of harassment and they express the clear wish for more information and counselling at MHH. A guideline for responding to sexual discrimination and violence was adopted by MHH in 2017.

In view of the complex situation regarding sexual harassment, the survey also highlights the role of patients. The results show that, even if fewer in number, repeated incidents involving patients occur. In past years, many undergraduate medical programs in Germany have given more attention to doctor/patient communication in the medical curriculum. How to handle cases in which patients clearly cross lines should also be systematically included in the issue of sexual harassment and discrimination [27].

As a result of this survey, the student initiative formed a project group which has reinforced gender awareness within the student community and on the campus. A series of formal and informal events and discussions was launched and used to promote official support in cases of sexual discrimination. In addition, workshops were designed and a reporting platform created. The workshops (15–20 participants) are directed toward teachers and students alike, following the motto of “teach the teacher”. A central workshop element is the re-enactment of reported harassment cases that are then discussed among the participants. The basis of the workshop is an open exchange among participants in order to mutually raise sensitivity to and awareness of the topic and to encourage reflexivity in daily routines. Initially guided by experts from the Netherlands, this program is designed to run independently in the future.

The reporting platform “#SayIt” was inspired by similar platforms in Berlin and the Netherlands. In 2019 it was made available to MHH students on the official MHH website [39]. The plan is to open the platform to all doctors as well. Like the workshops, this serves to increase the awareness of the everyday dimension and social practice of sexual discrimination that is deeply rooted in our “gender routines” –not just among men and women, but also within the gender boundaries that shape our identities. The ultimate goal of this anonymous reporting platform is to break the silence which too often goes hand-in-hand with incidents of discrimination. Too often there is the impression that acts of humiliation, in its various forms, are merely isolated events – not meant to harm or just meant to be “a joke”. Platform submissions will be published on a website if the reporting individual gives explicit permission. By posting the comments, everyone will be able to see that it is not an isolated case but rather a structural problem which is densely interwoven with our learning and working environments and one that deeply affects our privacy.

### Limitations and outlook

The response rate of approximately 15% might underestimate the actual willingness to participate since the email list also included inactive students and those on leave of absence who were unlikely to access their email accounts. Due to the relatively low response rate, we made an attempt to interpret the results conservatively even though the tendencies usually appeared to be clear. Otherwise, the facts that the share of men and women who responded to the survey is representative and that the share of people who indicated no sexual harassment is similar to other studies make us believe that these results are not biased by a stronger selection of students who were affected.

Additional studies should be undertaken at other German medical schools for the purpose of expanding on the findings of this study. Widening the focus on this topic would also be valuable. From a theoretical standpoint a more precise definition of the categories would be useful to better delineate the individual interpretations of “sexual discrimination” and “sexual harassment” and to more clearly identify connections with routine behaviour and everyday experiences.

How frequently discrimination occurs should be empirically documented with more precision, whether it was reported and which responses or measures followed. In the past, important cause/effect relationships that occurred in the course of discrimination have been portrayed primarily in qualitative interviews [10–12, 25].

## Conclusions

In conclusion, this single-centre study shows that sexual discrimination in undergraduate medical education exists and affects the attitudes and future career paths of medical students – predominantly women. Hence, experiences of sexism should not remain ignored any longer in empirical education research. In addition to ensuring the empirical basis, it would be ideal if methods of investigation were applied to reveal how gender-specific inequalities and discrimination in the medical profession can be effectively curtailed. More light should therefore be cast on the blind spot inhabited by sexual discrimination in medical education in Germany.

## Data Availability

The datasets analysed during the current study are available from the corresponding author on reasonable request.

## Abbreviations

AGG: Allgemeines Gleichstellungsgesetz (German anti-discrimination act)
AStA: Allgemeiner Studentenausschuss (Student Council)
DZHW: *Deutsches Zentrum für Hochschul- und Wissenschaftsforschung*
MHH: Medizinische Hochschule Hannover (Hannover Medical School)
TAN: Transaction number
